# Impact of Seasonal Nirsevimab Administration in Infants Born During the RSV Circulation Period on RSV-Related Hospitalizations: A Population-Based Study from Emilia-Romagna, Northern Italy

**DOI:** 10.3390/vaccines14020182

**Published:** 2026-02-14

**Authors:** Susanna Esposito, Matteo Puntoni, Giuseppe Maglietta, Alessandro De Fanti, Chiara Ghizzi, Giacomo Biasucci, Federico Marchetti, Melodie Olivia Aricò, Gianluca Vergine, Marcello Stella, Battista Guidi, Agnese Suppiej, Francesca Alberghi, Emanuele Filice, Maria Elena Capra, Enrico Valletta, Andrea Miceli, Cristina Malaventura, Beatrice Rita Campana, Valentina Fainardi, Caterina Caminiti, Emilia-Romagna Paediatric COVID-19 Network

**Affiliations:** 1 Pediatric Clinic, University-Hospital of Parma, 43126 Parma, Italy; beatricerita.campana@unipr.it (B.R.C.); valentina.fainardi@unipr.it (V.F.); 2 Department of Medicine and Surgery, University of Parma, 43126 Parma, Italy; giacomo.biasucci@unipr.it (G.B.); m.capra@ausl.pc.it (M.E.C.); 3 Clinical and Epidemiological Research Unit, University-Hospital of Parma, 43126 Parma, Italy; mpuntoni@ao.pr.it (M.P.); gmaglietta@ao.pr.it (G.M.); ccaminiti@ao.pr.it (C.C.); 4 Paediatrics Unit, Santa Maria Nuova Hospital, AUSL-IRCCS of Reggio Emilia, 42123 Reggio Emilia, Italy; alessandro.defanti@ausl.re.it (A.D.F.); francesca.alberghi@ausl.re.it (F.A.); 5 Paediatrics Unit, Maggiore Hospital, 40133 Bologna, Italy; chiara.ghizzi@ausl.bologna.it (C.G.); emanuele.filice@ausl.bologna.it (E.F.); 6 Pediatrics and Neonatology Unit, Guglielmo da Saliceto Hospital, 29122 Piacenza, Italy; 7 Pediatrics and Neonatology Unit, Ravenna Hospital, AUSL Romagna, 48121 Ravenna, Italy; federico.marchetti@auslromagna.it; 8 Pediatric Unit, G.B. Morgagni-L. Pierantoni Hospital, AUSL Romagna, 47121 Forlì, Italy; melodieolivialoredanarosa.arico@auslromagna.it (M.O.A.); enrico.valletta@auslromagna.it (E.V.); 9 Pediatric Clinic, Rimini Hospital, AUSL Romagna, 47923 Rimini, Italy; gianluca.vergine@auslromagna.it; 10 Pediatric Unit, AUSL Romagna, 47521 Cesena, Italy; marcello.stella@auslromagna.it; 11 Pediatric Unit, Pavullo Hospital, AUSL Modena, 41026 Pavullo, Italy; b.guidi@ausl.mo.it (B.G.); a.miceli@ausl.mo.it (A.M.); 12 Pediatric Clinic, University of Ferrara, 44124 Ferrara, Italy; agnese.suppiej@unife.it (A.S.); cristina.malaventura@unife.it (C.M.)

**Keywords:** respiratory syncytial virus, nirsevimab, monoclonal antibodies, immunoprophylaxis, RSV prevention, pediatric infectious diseases

## Abstract

Background: Respiratory syncytial virus (RSV) is a leading cause of hospitalization in early infancy, with the greatest burden occurring in the first months of life. Following the COVID-19 pandemic, many countries experienced intensified RSV circulation. Nirsevimab, a long-acting monoclonal antibody providing season-long protection after a single dose, was introduced in Italy for the 2024–2025 RSV season and recommended for infants born during the period of RSV circulation. We evaluated the population-level impact of this seasonal nirsevimab strategy on RSV-related hospitalizations among young infants. Methods: We conducted a population-based observational study using regional hospital discharge records from Emilia-Romagna, Northern Italy, spanning January 2017 to April 2025. Analyses were restricted to RSV seasons (October–March) and infants aged ≤180 days. RSV-related hospitalizations were identified using ICD-9-CM codes. Hospitalization rates were calculated per 100,000 person-days. Incidence rate ratios (IRRs) were estimated using negative binomial regression models adjusted for season, age group, and sex, with clustering at the hospital level. The post-nirsevimab season (2024–2025) was compared with the immediate pre-nirsevimab season (2023–2024) and a pre-COVID reference season (2018–2019). Results: A total of 551 RSV hospitalizations occurred in the pre-COVID season, 753 in the pre-nirsevimab season, and 252 in the post-nirsevimab season. The post-nirsevimab season was associated with a substantial reduction in RSV-related hospitalization rates compared with both the pre-COVID season (IRR 0.52; 95% CI 0.41–0.66) and the pre-nirsevimab season (IRR 0.36; 95% CI 0.29–0.44). Reductions were observed consistently across epidemic months and were most pronounced during the first three to four months of life. Conclusions: Seasonal administration of nirsevimab to infants born during the RSV circulation period was associated with a marked and sustained reduction in RSV-related hospitalizations in early infancy. These findings support the effectiveness of targeted, seasonally timed infant immunoprophylaxis as a population-level RSV prevention strategy.

## 1. Introduction

Respiratory syncytial virus (RSV) is a leading cause of acute lower respiratory tract infection in infants and young children worldwide and represents a major driver of paediatric hospitalizations during the winter season [1,2,3]. The burden of RSV disease is particularly high in the first months of life, when immunological immaturity, small airway caliber, and limited pre-existing immunity increase the risk of severe clinical manifestations such as bronchiolitis and pneumonia. Infants younger than six months account for a disproportionate share of RSV-related hospital admissions, healthcare utilization, and associated costs, even in high-income settings [4,5,6].

Until recently, options for RSV prevention in infancy were limited. Palivizumab, a monoclonal antibody requiring monthly administrations throughout the RSV season, has been restricted to a small subset of high-risk infants because of its cost and logistical constraints, leaving the majority of infants unprotected [7]. Consequently, RSV prevention at the population level remained an unmet public health need. This vulnerability became even more evident following the COVID-19 pandemic, which profoundly disrupted RSV epidemiology. Several countries reported atypical seasonal patterns and, once non-pharmaceutical interventions were lifted, intensified RSV circulation with increased hospitalization rates among infants and young children [6].

Nirsevimab, a long-acting monoclonal antibody targeting the prefusion F protein of RSV, represents a major advance in RSV prevention [7,8,9,10]. A single intramuscular dose provides protection for an entire RSV season and is indicated for infants entering their first RSV season, regardless of underlying risk conditions. Randomized clinical trials and early real-world studies have demonstrated high efficacy and effectiveness of nirsevimab in preventing RSV-related lower respiratory tract infection and hospitalization when administered to infants before or during the RSV season [11,12,13,14,15,16,17,18,19,20,21,22,23,24,25,26]. However, despite its clinical promise, concerns regarding costs and implementation logistics have led some health systems to adopt targeted or seasonal administration strategies rather than universal year-round use.

In Italy, nirsevimab was introduced into routine clinical practice for the 2024–2025 RSV season, with regional variability in recommendations and implementation [27]. In the Emilia-Romagna Region, nirsevimab was recommended for all infants born between October 2024 and March 2025—corresponding to the period of RSV circulation—as well as for selected high-risk children in their second year of life [28]. Evaluating the real-world impact of this seasonally targeted strategy is essential to inform public health decision-making, particularly in the context of post-pandemic RSV resurgence and ongoing debates about universal versus selective prophylaxis.

The aim of this population-based study was to assess the impact of nirsevimab introduction on RSV-related hospitalizations among infants aged 0–180 days in the Emilia-Romagna Region, Northern Italy. By comparing hospitalization rates across a pre-pandemic season, the immediate pre-nirsevimab season, and the first post-nirsevimab season, and by exploring age-specific and birth cohort-based patterns, we sought to quantify changes in RSV hospitalization risk and to characterize the temporal persistence of protection during the first months of life.

## 2. Methods

### 2.1. Study Design and Population

We conducted a population-based observational study using monthly RSV hospitalization data extracted from regional hospital discharge records covering the period from January 2017 to March 2025. Analyses were restricted to infants aged ≤180 days (0–6 months), corresponding to the population eligible for nirsevimab administration during the RSV season in the 2024–2025 period (October 2024–March 2025).

In the Emilia-Romagna Region, RSV circulation has historically shown a consistent seasonal pattern, with the majority of laboratory-confirmed infections and RSV-related hospitalizations occurring between October and March. Regional surveillance data and hospital discharge trends indicate that, despite post-COVID-19 disruptions observed in some settings, RSV activity during the three study seasons considered (2018–2019, 2023–2024, and 2024–2025) remained largely encompassed within this October–March timeframe. In particular, RSV-related hospitalizations during each season showed a progressive increase from late autumn, peaked during the winter months (December–February), and declined by March, with minimal activity observed outside this interval. Therefore, restricting the analyses to October–March ensured comprehensive capture of the RSV epidemic period across all study seasons while maintaining consistency in seasonal comparisons and avoiding dilution of estimates by months with negligible RSV circulation.

During the 2024–2025 RSV season, nirsevimab was implemented in the Emilia-Romagna Region through a centrally coordinated regional program targeting all eligible infants born during the RSV circulation period. Regional monitoring data indicated high uptake, with coverage exceeding 85% among eligible newborns, and no relevant differences observed across hospitals or months of the season. Administration was routinely performed immediately after birth (age range, 1–7 days of life; median, 3), before hospital discharge, according to standardized regional protocols, minimizing delays and ensuring early protection during the highest-risk period of life. No major logistical or organizational challenges affecting rollout or access were reported. Individual-level data on nirsevimab administration were not available for linkage with hospitalization records. Although the maternal RSV prefusion F (RSVpreF) vaccine was approved and commercially available in Italy during the 2024–2025 season, it was not implemented as part of a regional maternal immunization program in Emilia-Romagna during the study period; therefore, maternal RSV vaccination uptake was negligible and was not expected to confound the study findings.

Age at hospital admission was available in days and categorized into six predefined 30-day age bands (0–29, 30–59, 60–89, 90–119, 120–149, and 150–180 days), allowing detailed age-specific analyses during early infancy. Hospitalizations occurring after 180 days of age were excluded, as they fell outside the target age window for RSV immunoprophylaxis and the study objective.

The study included hospitalizations recorded at the following centers: Parma University Hospital, Piacenza Hospital, Reggio Emilia Hospital, Bologna Maggiore Hospital, Pavullo Hospital, Ferrara Hospital, Forlì Hospital, Cesena Hospital, Rimini Hospital, and Ravenna Hospital. Together, these institutions accounted for approximately 50% of all hospitalizations among infants aged <6 months in the Emilia-Romagna Region, Northern Italy, ensuring substantial coverage and geographic representativeness of the regional pediatric population.

### 2.2. Case Definition

RSV-related hospitalizations were identified based on the presence of one or more relevant ICD-9-CM diagnosis codes recorded in any diagnostic position. These included bronchiolitis due to RSV (466.11), pneumonia due to RSV (480.1), RSV infection (079.6), bronchiolitis not otherwise specified (466.19), pneumonia not otherwise specified (485), and bronchopneumonia (486).

This inclusive case definition was adopted to capture both laboratory-confirmed RSV infections and clinically compatible lower respiratory tract infections, reflecting routine diagnostic practices in early infancy where virological confirmation may not be systematically performed and to minimize potential bias related to temporal changes in RSV testing and diagnostic coding practices across seasons, particularly in the post-pandemic period. Only hospitalizations meeting this case definition were included in the analyses.

### 2.3. Data Aggregation and Study Periods

Hospitalization counts were aggregated at the monthly level by age band, sex, and hospital center. To ensure compatibility with count-based regression models, strata with zero observed events were retained in the dataset.

Analyses were restricted to the RSV season, defined as October through March, for each study year. Three RSV seasons were defined a priori. The primary comparison focused on the 2024–2025 season, corresponding to the first RSV season following the introduction of nirsevimab, and the 2023–2024 season, representing the immediate pre-nirsevimab period. Additionally, the 2018–2019 RSV season was included as a pre-pandemic reference, providing contextual comparison with a season unaffected by COVID-19–related disruptions in RSV circulation, healthcare-seeking behavior, or hospital admission practices.

### 2.4. Hospitalization Rates and Person-Time Estimation

Hospitalization rates were calculated as the number of RSV-related admissions divided by the estimated person-time at risk and expressed per 100,000 person-days. In the absence of monthly birth counts, person-time was approximated assuming a uniform distribution of births across the calendar year.

For season-level analyses, cumulative person-time was assumed to increase progressively from October (corresponding approximately to 0–1 months of age) through March (corresponding to 0–6 months of age), reflecting the accumulation of infants entering the age window over the course of the RSV season.

For regression analyses stratified by age band, person-time denominators were defined according to age in days rather than calendar time. Specifically, for each 30-day age band, the offset was calculated as the annual population aged 0 years divided by 365.25 and multiplied by the width of the age band. This approach provided an approximation of the number of person-days at risk within each age category and allowed consistent comparison across seasons.

### 2.5. Statistical Analysis

Incidence rate ratios (IRRs) were estimated using count regression models with a log(person-time) offset. For season-level analyses, negative binomial regression models were used to account for overdispersion in hospitalisation counts. For analyses based on birth cohorts, where no evidence of overdispersion was observed and sparse month-specific cells were present, Poisson regression models with robust standard errors were used to obtain stable inference. Models included season, age at admission, and sex as covariates and robust standard errors were clustered at the hospital centre level to account for within-centre correlation. In analyses based on birth cohorts, age was modelled as months since birth and included as a continuous variable interacting with season to avoid model saturation due to sparse month-specific cells. This modelling approach yields relative effect estimates (IRRs) that are not directly comparable with crude age-specific rates, but rather quantify season-specific differences in risk conditional on age since birth.

The primary parameter of interest was the adjusted IRR comparing the 2024–2025 (post-nirsevimab) season with the 2023–2024 (pre-nirsevimab) season. Comparisons with the 2018–2019 RSV season were treated as secondary, contextual analyses aimed at assessing the robustness of findings relative to a pre-pandemic baseline.

## 3. Results

Table 1 summarizes the characteristics of RSV-related hospitalizations among infants aged 0–180 days across three consecutive seasons: the pre-COVID season (1 October 2018–31 March 2019), the pre-nirsevimab season (1 October 2023–31 March 2024), and the post-nirsevimab season (1 October 2024–31 March 2025). A total of 551 RSV hospitalizations were recorded in the pre-COVID season, increasing to 753 in the pre-nirsevimab season, and then markedly decreasing to 252 in the post-nirsevimab season. Across all seasons, hospitalizations were more frequent in males than females, with a stable sex distribution of approximately 58% males and 41% females. Regarding age distribution, hospitalizations were most common in the first two months of life in all periods, particularly among infants aged 30–59 days. In the post-nirsevimab season, a relative shift toward older age groups was observed, with higher proportions of hospitalizations among infants aged 120–149 days and 150–180 days compared with earlier seasons. The hospitalization rate showed substantial variation across seasons. It was 32.2 per population unit (95% CI: 29.6–35.0) in the pre-COVID season, increased to 48.2 (95% CI: 44.8–51.8) in the pre-nirsevimab season, and then declined sharply to 16.7 (95% CI: 14.7–18.9) in the post-nirsevimab season. Overall, the table highlights a pronounced reduction in RSV hospitalization burden following the introduction of nirsevimab, with consistent sex distribution and notable changes in age-specific patterns.

**Figure 1:**
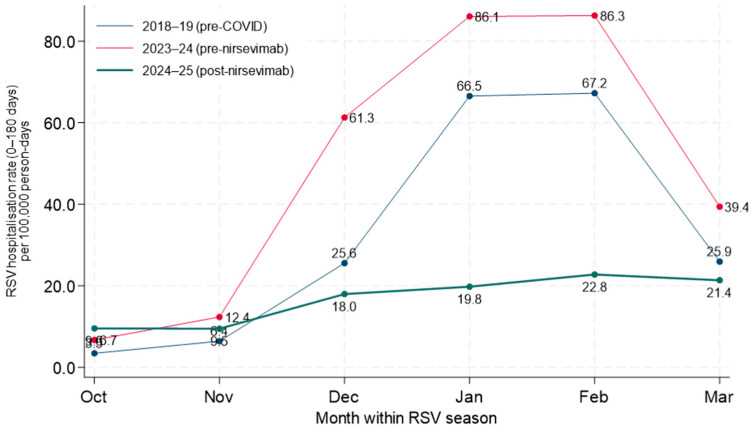
Analysis of RSV hospitalization rates aggregated at the seasonal level, adjusted for sex and age.

In the monthly comparison within the RSV season (Figure 1), hospitalization rates among infants aged 0–180 days showed a marked seasonal increase during the pre-nirsevimab seasons, with values rising progressively from the beginning of the season and peaking between January and February, followed by a decline in March. In contrast, during the post-nirsevimab season, rates are consistently lower across all months, with a substantial attenuation of the winter peak and a flatter temporal profile, characterized by limited variability and a gradual decrease toward the end of the season.

In the graph, each point represents the monthly RSV hospitalization rate among infants aged 0–180 days hospitalized in the specific month of the epidemic season shown on the *x*-axis. For each month and season, the numerator consists of the number of RSV hospitalizations observed in that month, while the denominator is the cumulative person-time at risk, expressed in person-days, estimated from the resident population aged under one year and restricted to the 0–180-day age interval.

In the multivariable model (Table 2), the post-nirsevimab season (2024–2025) was associated with a marked and statistically significant reduction in the RSV hospitalization rate among infants aged 0–180 days, both compared with the pre-COVID 2018–2019 season (IRR 0.52; 95% CI 0.41–0.66) and, even more pronouncedly, with the season immediately preceding the introduction of nirsevimab (2023–2024; IRR 0.36; 95% CI 0.29–0.44). In contrast, the 2023–2024 season showed a significant increase in risk compared with the pre-pandemic period (IRR 1.46; 95% CI 1.20–1.79), confirming intensified RSV circulation in the post-pandemic period in the absence of prophylaxis. With regard to age, the risk of RSV hospitalization followed a non-monotonic pattern in the first months of life: compared with neonates aged 0–29 days, the risk was significantly higher in the 30–59-day age group (IRR 1.490; 95% CI 1.27–1.75) and remained elevated, though to a lesser extent, in the 60–89-day age group (IRR 1.13; 95% CI 0.97–1.32), followed by a progressive and significant reduction in risk in the subsequent age groups (≥90 days). Finally, female sex was associated with a significantly lower risk of hospitalization compared with male sex (IRR 0.71; 95% CI 0.64–0.79).

**Figure 2:**
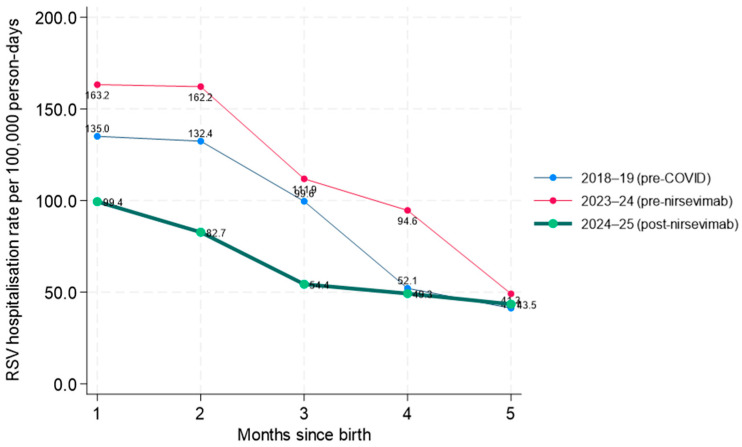
Secondary analysis based on birth cohorts (as a proxy for month of nirsevimab administration), aimed at exploring trends in hospitalization rates in the months following birth and comparison with the pre-nirsevimab seasons.

Across all seasons, the risk of hospitalization was highest in the first two months of life and decreased progressively with increasing age (Figure 2). In the post-nirsevimab season (2024–2025), hospitalization rates were consistently lower than in previous seasons in each month of follow-up. However, differences between seasons attenuated with increasing age. In particular, at four months of age, hospitalization rates in the post-nirsevimab and pre-COVID seasons appeared similar, and by five months of age the rates were nearly overlapping across all three seasons, indicating convergence of absolute risks at older ages.

Each point in the graph represents the RSV hospitalization rate, expressed per 100,000 person-days, observed in a specific birth cohort (month of birth ≈ month of nirsevimab administration, from November to March) at a given number of months since birth (*x*-axis). For each value of t (months since birth), the analysis includes only hospitalizations observed among children belonging to the birth cohort under consideration who had reached that age at the time of the event. Person-time denominators are therefore cohort- and follow-up-specific, excluding periods that were not observable or that occurred before the birth of the cohort.

Table 3 presents adjusted incidence rate ratios derived from a birth cohort-based Poisson regression model, comparing RSV hospitalization risk in the post-nirsevimab season, with previous seasons at specific months since birth. Compared with the pre-COVID season (2018–2019), the post-nirsevimab season (2024–2025) was associated with a significantly lower risk of hospitalization during the first four months of life, whereas by the fifth month the difference was no longer statistically significant. In contrast, when compared with the immediate pre-nirsevimab season (2023–2024), a significantly lower risk persisted across all months of follow-up, including the fifth month.

## 4. Discussion

In this population-based study, the introduction of nirsevimab administered to infants born during the RSV circulation season was associated with a marked and statistically significant reduction in RSV-related hospitalizations among infants aged 0–180 days. By comparing the first post-nirsevimab season (2024–2025) with both the immediately preceding season and a pre-pandemic reference season, our analysis provides robust real-world evidence supporting the effectiveness of nirsevimab when implemented at scale in routine clinical practice under a seasonally targeted strategy.

The magnitude of the reduction observed in our setting is substantial. Compared with the 2023–2024 season, which immediately preceded nirsevimab introduction, RSV hospitalization rates declined by approximately 64%, while a reduction of nearly 50% was observed relative to the pre-COVID 2018–2019 season. These population-level estimates are remarkably consistent with results from pivotal randomized controlled trials. In the MELODY and HARMONIE studies, nirsevimab demonstrated efficacy ranging from 74% to over 80% against RSV-related lower respiratory tract infection requiring medical attention, with reductions of approximately 75% in hospitalization risk [29,30]. Although direct comparisons between trial efficacy and real-world effectiveness should be interpreted with caution, the close convergence of these estimates strengthens confidence in the robustness, generalizability, and external validity of the observed effect. The slightly lower magnitude of effectiveness observed in our study compared with estimates from randomized trials and some laboratory-confirmed real-world studies may, at least in part, be explained by the case definition adopted. RSV-related hospitalizations were identified using a broad set of ICD-9-CM codes encompassing both RSV-specific diagnoses and clinically compatible lower respiratory tract infections, reflecting routine clinical practice in early infancy, where systematic viral testing is not uniformly performed. Consequently, some hospitalizations included in our outcome definition may have been caused by respiratory pathogens other than RSV, leading to a non-differential misclassification of the outcome. This type of misclassification would be expected to bias effect estimates toward the null, thereby underestimating the true preventive effect of nirsevimab. Importantly, the same case definition was applied consistently across all study seasons, ensuring internal validity of relative comparisons. From a public health perspective, this pragmatic approach captures the real-world burden of severe bronchiolitis requiring hospitalization and provides conservative, policy-relevant estimates of impact under routine care conditions.

Our findings are also consistent with emerging real-world evidence from early-adopting countries and from Italian regions that implemented universal nirsevimab administration strategies [12,13,14,15,16,17,18,19,20,21,22,23,24,25,26]. Surveillance data from France and Spain, where nirsevimab was broadly deployed during the 2023–2024 season, have reported sharp declines in RSV-associated hospitalizations among infants compared with previous years, particularly during peak winter months. These reports suggest a substantial blunting of the RSV epidemic curve following widespread immunoprophylaxis. The pronounced attenuation of the seasonal peak observed in our monthly analyses closely mirrors these experiences and underscores the potential of nirsevimab not only to reduce individual-level risk but also to mitigate seasonal healthcare system burden and epidemic intensity, even when implemented in a targeted, seasonal manner.

Although the observed reduction in RSV-related hospitalizations following nirsevimab implementation is substantial and consistent across analytical approaches, the observational design of this study precludes definitive causal inference. Accordingly, our findings should be interpreted as demonstrating a strong temporal and population-level association between nirsevimab introduction and reduced RSV hospitalization burden, rather than establishing causality in the strict experimental sense. Nevertheless, the magnitude, consistency, and biological plausibility of the observed associations—together with their alignment with randomized trial efficacy and early real-world effectiveness data—support the interpretation that nirsevimab contributed meaningfully to the decline in hospitalizations under routine care conditions. Importantly, our estimates reflect real-world effectiveness in a pragmatic setting, integrating implementation factors such as high but not universal coverage, routine clinical case definitions, and population heterogeneity, and therefore provide complementary evidence to efficacy estimates derived from controlled trial environments.

The age-specific patterns observed in our study further support the biological plausibility of the findings. Across all seasons, RSV hospitalization risk was highest during the first two months of life, consistent with extensive literature documenting the particular vulnerability of neonates and young infants to severe RSV disease [4,5,6,31]. Importantly, during the post-nirsevimab season, hospitalization rates were consistently lower across all months of follow-up, with statistically significant reductions persisting through the third and fourth months of life. This temporal pattern closely reflects the pharmacokinetic profile of nirsevimab, which maintains protective antibody concentrations for at least five months following a single dose. The gradual attenuation of protection beyond the fourth month is also consistent with trial data demonstrating declining, yet still clinically meaningful, efficacy toward the end of the RSV season [32,33].

The secondary birth-cohort analysis provides additional methodological strength and complements the season-level comparisons. By aligning follow-up time with age since birth and approximating the timing of nirsevimab administration, this approach reduces confounding related to calendar time, epidemic phase, and RSV circulation intensity. The consistency of results across multiple analytical strategies supports a causal interpretation and reduces the likelihood that the observed reductions were driven by secular trends, changes in admission thresholds, or shifts in healthcare-seeking behavior.

The increased RSV hospitalization risk observed during the 2023–2024 season compared with the pre-pandemic period deserves particular attention. This finding corroborates reports from multiple countries describing intensified RSV epidemics following the COVID-19 pandemic [6]. Proposed explanations include immunity debt due to reduced viral exposure during lockdowns, altered maternal antibody transfer, and changes in social mixing patterns. Against this backdrop of heightened baseline risk, the sharp and sustained decline observed after nirsevimab introduction is especially compelling and suggests that infant immunoprophylaxis effectively counteracted the post-pandemic resurgence of RSV.

Sex-related differences in hospitalization risk, with females consistently exhibiting a lower risk than males, align with prior epidemiological evidence and are likely attributable to sex-based differences in lung development, immune responses, and susceptibility to severe respiratory infections [34]. The persistence of this association across seasons further supports the internal consistency and validity of our findings.

This study has several strengths, including large population coverage, inclusion of multiple RSV seasons spanning pre-pandemic and post-pandemic periods, and the use of robust statistical methods accounting for overdispersion and clustering at the hospital level. Nonetheless, some limitations should be acknowledged. RSV case identification relied on administrative diagnostic codes rather than systematic laboratory confirmation; although this approach is widely used in large-scale surveillance studies and was applied consistently across seasons, it may have resulted in non-differential outcome misclassification and conservative effect estimates. The use of a broad case definition was intentionally chosen to ensure comparability across seasons and avoid sparse data, particularly in month-specific birth cohort analyses; restricting analyses to RSV-specific ICD-9-CM codes could also have introduced differential ascertainment given changes in RSV testing practices over time, especially in the post-pandemic period. Individual-level data on nirsevimab administration were not available, precluding direct estimation of effectiveness and limiting inference to population-level impact. In addition, although models were adjusted for age, sex, and season, adjustment for other potentially relevant confounders—including gestational age—was not feasible due to the absence of individual-level perinatal data in the hospitalization records. Consequently, residual confounding related to prematurity and other perinatal factors cannot be excluded. Further potential sources of residual confounding include post-pandemic changes in healthcare-seeking behavior, evolving hospital admission thresholds, co-circulation of other respiratory viruses, and temporal variability in RSV testing practices. Assumptions regarding uniform birth distribution may also have introduced minor imprecision in person-time estimation, although such bias is unlikely to account for the magnitude or consistency of the observed reductions. Finally, the relative shift toward older age at hospitalization observed in the post-nirsevimab season warrants further investigation. This pattern may reflect sustained protection during the first months of life with gradual attenuation over time, consistent with the known pharmacokinetic profile of nirsevimab and observations from other European settings; however, our study was not designed to formally assess waning protection or age-specific differences in disease severity, and such data were not available.

The generalizability of our findings should be considered in light of contextual differences across settings. Emilia-Romagna is characterized by a universal, publicly funded healthcare system with high hospital accessibility, standardized neonatal care pathways, and centralized coordination of immunoprophylaxis programs, which likely facilitated the high and homogeneous uptake of nirsevimab observed in this study. Regions with fragmented healthcare systems, lower access to perinatal care, or delayed postnatal follow-up may experience different levels of coverage and timing of administration, potentially attenuating population-level impact. Moreover, RSV seasonality in Emilia-Romagna remains relatively well defined and largely confined to the winter months; in settings with prolonged, irregular, or year-round RSV circulation, the effectiveness of a seasonally targeted strategy may differ and require alternative timing or broader implementation. Nevertheless, the consistency of our findings with early post-implementation data from other European regions suggests that substantial reductions in RSV-related hospitalizations are achievable across diverse contexts when high coverage and timely administration are ensured.

## 5. Conclusions

Our findings add to the growing body of evidence demonstrating that nirsevimab, even when administered exclusively to infants born during the RSV circulation season, substantially reduces RSV-related hospitalizations in early infancy under real-world conditions. The consistency of our results with evidence from settings that adopted universal nirsevimab strategies supports infant immunoprophylaxis as a cornerstone of RSV prevention. Ongoing surveillance will be essential to assess the long-term impact of seasonal strategies, durability of protection, potential shifts in RSV epidemiology, and interactions with emerging maternal RSV vaccination programs.

## Figures and Tables

**Table 1:** Characteristics of RSV hospitalisations (0–180 days) by season.

	Pre-COVID (1 October 2018–31 March 2019)	Pre-Nirsevimab (1 October 2023–31 March 2024)	Post-Nirsevimab (1 October 2024–31 March 2025)
RSV hospitalizations	N = 551	N = 753	N = 252
Sex, n (%)			
Male	324 (58.8%)	437 (58.0%)	150 (59.5%)
Female	227 (41.2%)	316 (42.0%)	102 (40.5%)
Age group, n (%)			
0–29 days	108 (19.6%)	127 (16.9%)	36 (14.3%)
30–59 days	143 (26.0%)	203 (27.0%)	63 (25.0%)
60–89 days	122 (22.1%)	159 (21.1%)	36 (14.3%)
90–119 days	83 (15.1%)	110 (14.6%)	28 (11.1%)
120–149 days	46 (8.3%)	101 (13.4%)	47 (18.7%)
150–180 days	49 (8.9%)	53 (7.0%)	42 (16.7%)
Hospitalization rate (95%CI) *	32.2 (29.6–35.0)	48.2 (44.8–51.8)	16.7 (14.7–18.9)

* Rates expressed per 100,000 person-days. Confidence intervals were calculated assuming a Poisson distribution of the observed counts.

**Table 2:** Negative binomial regression of RSV hospitalisations (0–180 days).

	IRR	95% CI	*p*-Value
Season			
Pre-Nirsevimab (2023–24) vs. Pre-COVID (2018–19)	1.46	1.20–1.79	<0.001
Post-Nirsevimab (2024–25) vs. Pre-COVID (2018–19)	0.52	0.41–0.66	<0.001
Post-Nirsevimab (2024–25) vs. Pre-Nirsevimab (2023–24)	0.36	0.29–0.44	<0.001
Sex			
Male	1.00		
Female	0.71	0.64–0.79	<0.001
Age group (days)			
0–29	1.00		
30–59	1.49	1.27–1.75	<0.001
60–89	1.13	0.97–1.32	0.107
90–119	0.79	0.76–0.82	<0.001
120–149	0.76	0.65–0.89	<0.001
150–180	0.57	0.51–0.64	<0.001

IRR: Incidence Rate Ratios; 95%CI: 95% confidence interval.

**Table 3:** Incidence rate ratios (IRRs) of RSV hospitalisation by month since birth, estimated using Poisson regression: contrasts between post-nirsevimab (2024–2025) and pre-nirsevimab (2023–2024) and pre-COVID (2018–2019) seasons.

	Post vs. Pre-COVID (2018–19)		Post vs. Pre-Nirsevimab (2023–24)	
Months Since Birth	IRR (95% CI)	*p*-Value	IRR (95% CI)	*p*-Value
1	0.68 (0.57–0.80)	<0.001	0.57 (0.49–0.66)	<0.001
2	0.69 (0.60–0.79)	<0.001	0.56 (0.49–0.63)	<0.001
3	0.70 (0.58–0.84)	<0.001	0.54 (0.47–0.63)	<0.001
4	0.71 (0.54–0.93)	0.014	0.53 (0.43–0.65)	<0.001
5	0.72 (0.50–1.05)	0.087	0.52 (0.39–0.69)	<0.001

IRR: Incidence Rate Ratios; 95%CI: 95% confidence interval.

## Data Availability

The data presented in this study are available on request from the corresponding author due to due to privacy restrictions.
